# Lowering technical barriers in microbiology education using archaea: Halobacterium salinarum as a school laboratory model

**DOI:** 10.1099/acmi.0.001185.v3

**Published:** 2026-07-30

**Keywords:** crystal violet, education, *Halobacterium salinarum*, microbiology, school labs, sterile conditions

## Abstract

Traditional microbiology education is fundamentally constrained by the requirement for sterile laboratory conditions, which limits the implementation of practical activities in school settings. This limitation can be overcome by using *Halobacterium salinarum*, a halophilic archaeon that grows readily on media containing up to 5 mol·l⁻¹ (290 g·l⁻¹) sodium chloride, effectively preventing contamination. Moreover, this micro-organism is non-pathogenic and well suited for educational use.

This article presents two simple hands-on activities that can be easily carried out in a standard school science laboratory using common consumer products with the aim of reducing operational costs. The first activity introduces basic microbiological techniques by highlighting characteristic features of *H. salinarum* colonies, including their pink pigmentation and violet fluorescence under UV light. The second activity involves microscopic observation of cells stained with crystal violet, using microscope slides prepared according to a protocol adapted for halophilic microorganisms. Well-stained, flexible rod-shaped cells are observed, reflecting the absence of a rigid cell wall.

Following these activities, a short questionnaire is proposed to encourage reflection on the experimental work performed. Instructors are also invited to discuss with students the role of extremophiles in the evolution of life forms.

**Impact Statement**

This article is intended for high school science teachers wishing to implement fundamental microbiological techniques in practical classroom activities.

## Data Summary

The author confirms all supporting data, code and protocols have been provided within the article.

## Introduction

The teaching of microbiology may be problematic if its practical aspects cannot be performed in sterile environments. Most schools do not have a dedicated microbiology laboratory, and the handling of micro-organisms is usually done in a biology or a chemistry laboratory, which lacks any controlled environment. This situation makes contamination almost unavoidable, and practical microbiology activities are very limited.

Moreover, standard research microbiology laboratories are classified according to strict biosafety regulations [1, 2, 3], and the implementation of these standards entails significant constraints, which are impossible to meet in school science laboratories.

If it turns out that no biological control can be carried out, but the curriculum aims to teach the basics of practical microbiology, the following two conditions must at least be met:

1. Use non-pathogenic biological material
2. Avoid the need to work under sterile conditions, whilst adhering to good occupational safety and hygiene practices

In the search to find a solution to these issues, Professor S. DasSarma from the University of Maryland, USA, and his colleagues have pioneered the use of *Halobacterium salinarum* as a model organism in classrooms [4]. The advantages of working with this micro-organism are:

- Like all presently known archaea, it is not pathogenic.
- Due to its extremely halophilic (salt-loving) living conditions, the handling of *H. salinarum* does not require sterile conditions (e.g. autoclaving media and sterilizing utensils). A clean working place and the observance of good hygienic practices are sufficient for working with this micro-organism.

Since its first description in 1922, the peculiar biology of *H. salinarum* has been extensively documented [5]. The purpose of this article is to show that microbiology can be made simple while discovering new concepts in biology.

*H. salinarum* is an extremely halophilic micro-organism. It grows in very high saline environments in which the concentration of sodium chloride (NaCl) may go up to 5 mol⋅l^−1^ (290 g⋅l^−1^), which is close to saturation: at 20 °C, the solubility of NaCl is 358.5 g⋅l^−1^. *H. salinarum* is found in hyper-saline lakes and salterns (an installation or system of shallow ponds used to extract crystalline salt from saltwater) and sometimes in high-salt food [6] (e.g. ‘Bacalau’, salt-preserved cod). Due to their high salinity, these salterns develop a reddish or pink colour with the presence of halophilic Archaea and algae like *Dunaliella*. As a species that colonizes salterns, *H. salinarum* is known for its distinct colour and abundance at places where saline levels reach 4 mol⋅l^−1^ NaCl. The colour is due to the presence of *halorubin*, a carotenoid pigment.

*H. salinarum* is an obligate aerobe (needs oxygen to grow). However, when oxygen is depleted, as happens in salterns where temperature and cell densities are high (around 10^7^–10^8^ cells·ml^−1^), cellular energy production switches to *bacteriorhodopsin*, a purple photoactive enzyme (protein) present in cell membranes. Bacteriorhodopsin is similar to vertebrate *rhodopsins*, the pigments that sense light (photoreceptors) in the retina [7].

## Methods

### Activity 1: Culture and observation of *H. salinarum*

Enquire from your local school lab supplier if *H. salinarum* is available. In this case, the strain was purchased from Sordalab (France) https://sordalab.com/index.php. Strains can also be obtained from Carolina Biological Supply (USA, worldwide shipping available https://www.carolina.com/archaea-halobacterium/halobacterium-sp-tube/154800.pr). It is possible to isolate strains of *H. salinarum* from various habitats (solar salt pans, salted foods), but this requires preliminary characterisation work that goes beyond the scope of this basic educational activity.

Samples are usually delivered as colonies on a Petri dish or in a test tube. *H. salinarum* may be easily subcultured several times.

Culture media formulations:

For school labs, a formula that is easy to prepare has been created considering the use, when possible, of daily life ingredients and the growth performance on solid and liquid media. In the present case, prepare a ‘basal medium’: a medium containing water, a carbon source, a nitrogen source and various mineral salts but with a very high NaCl content. This study will focus on solid media. ‘Solid’ means that the medium, initially prepared as an aqueous solution, is gelled by addition of agar–agar, a polysaccharide of algal origin and subsequent heating and cooling. After being inoculated at the surface of the gelled medium, micro-organisms develop and are visualized by the appearance of ‘colonies’.

Keeping in mind the availability of chemicals/ingredients, microbiological grade powdered yeast extract and peptone have been replaced by similar ingredients obtained from local stores. These two ingredients are easily available in supermarkets, as well as deionized water.

Powdered agar is more difficult to obtain from supermarkets, but it can easily be found in drugstores or health food stores. However, this type of agar is less easy to dissolve than bacteriological grade agar.

Please note that NaCl (kitchen salt) and KCl (also available in shops for low-sodium diets) should be chosen pure, without any additives. Epsom salt is available in pharmacies. Na_3_citrate is a food additive and is used here as a pH buffer. Ideally, but not mandatory, the pH of this medium should be adjusted to 7.5 (slightly alkaline, aligned with seawater concentration). See composition in Table 1.

**Table 1.** Substances used for media preparation

Chemical/ingredient	Chemical formula	Common/brand name	Availability	Amount g⋅l^−1^	Molarity (mole⋅l^−1^)
Sodium chloride	NaCl	Table salt	Supermarkets	250	4.28
Magnesium sulphate heptahydrate	MgSO_4_·7H_2_O	Epsom salt	Pharmacies/chemist’s/drugstores	20	8.1⋅10^−2^
Trisodium citrate	Na_3_C_6_H_5_O_7_	na	Drugstores/chemical supply companies	3	1.2⋅10^−2^
Potassium chloride	KCl	na	Supermarkets/drugstores	2	2.6⋅10^−2^
Yeast extract	na	*Freeze-dried beer yeast (debittered) or Marmite^®^*	Supermarkets	5	na
Peptone	na	*Bovril^®^*	Supermarkets	5	na
Powdered agar	na	Agar–agar	Supermarkets	20	na
Water (deionized)	H_2_O	Water	Supermarkets	Up to 1 L	na

NA, not applicable.

Materials

Growth on solid media.

- 1-l graduated cylinder
- 1-l beaker or similar glassware
- Scale (0.1 g accuracy)
- Spoons or spatulas for weighing
- 1-l Erlenmeyer flask or similar glassware
- Magnetic stirrer with heating and magnetic rod
- A few plastic Petri dishes (Ø 88 mm is fine)
- Cotton buds
- A *H. salinarum* strain
- A binocular magnifier
- A handheld UV (black light) lamp

**Fig. 1.**
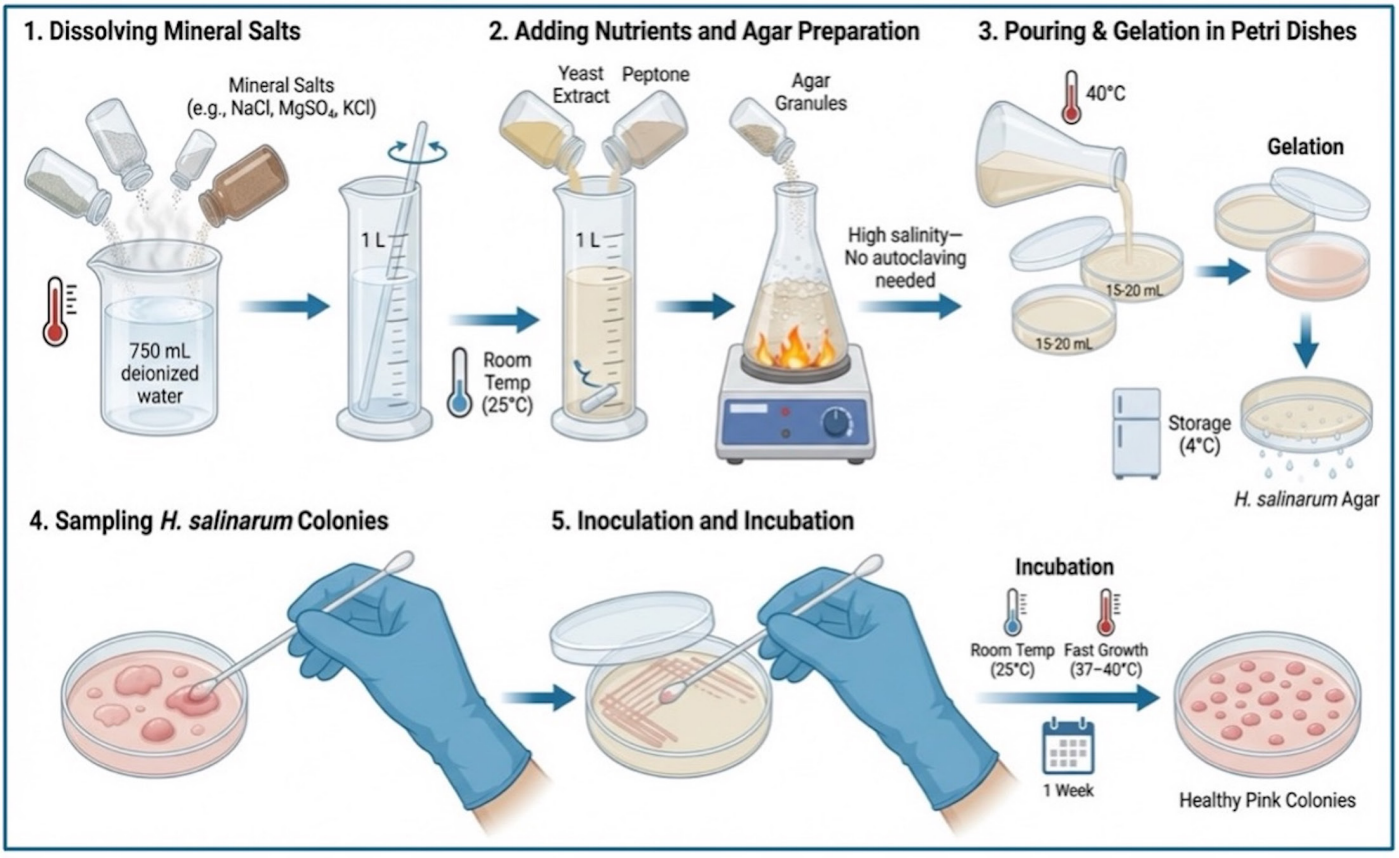
Illustrated *H. salinarum* culture protocol

Procedure (see Fig. 1.)

1. Completely dissolve the mineral salts in about 750 ml of hot deionized water in a glass container and pour the solution into the graduated cylinder. Allow to cool down to room temperature.

*2*. Add yeast extract and peptone to the cylinder and adjust to 1 l with deionized water; mix well. Agar won’t dissolve at room temperature; it is recommended to add the agar when the medium is brought to a boil in the Erlenmeyer flask. Mix well while boiling to avoid clogging. Because of its very high salinity, it is not necessary to autoclave this medium.

1. Let the medium cool to ~40 °C and pour about 15–20 ml into the Petri dishes. Put the lids back on and allow the medium to gel at room temperature. If no experiment is performed afterwards, the Petri dishes may be stored for several days in a refrigerator. It is preferred to store them upside down to control condensation.

1. Collect *H. salinarum* samples from the original Petri dish by gently rubbing the pink coloured colonies with a cotton bud (or an inoculation loop if available).
2. Inoculate the surface of the freshly prepared Petri dishes, put the lid back on and let them incubate at room temperature (~25 °C) for at least 1 week. Note: The ideal growth temperature is 37–40 °C. In these conditions, colonies grow much faster.
3. Once the colonies are well established, they can be observed with a binocular magnifier directly on the Petri dish.
4. Shine a UV light (395 nm) on the colonies to produce a purple fluorescence.

### Activity 2: *H. salinarum* under the microscope

The best way to visualize individual cells is – of course – under the microscope. Here is a simple protocol describing this activity.

Materials

- NaCl 20% aqueous solution
- Acetic acid 2% aqueous solution or alcohol vinegar (6°/8° acidity) diluted three-/fourfold in deionized water
- Crystal violet 0.25% aqueous solution for staining. Crystal violet staining constitutes part of the Gram staining protocol, which is a standard procedure in microbiology. It is readily available from chemical products companies.
- Distilled water
- Immersion oil (for microscopy)
- Plastic pipette
- Microscope

**Fig. 2.**
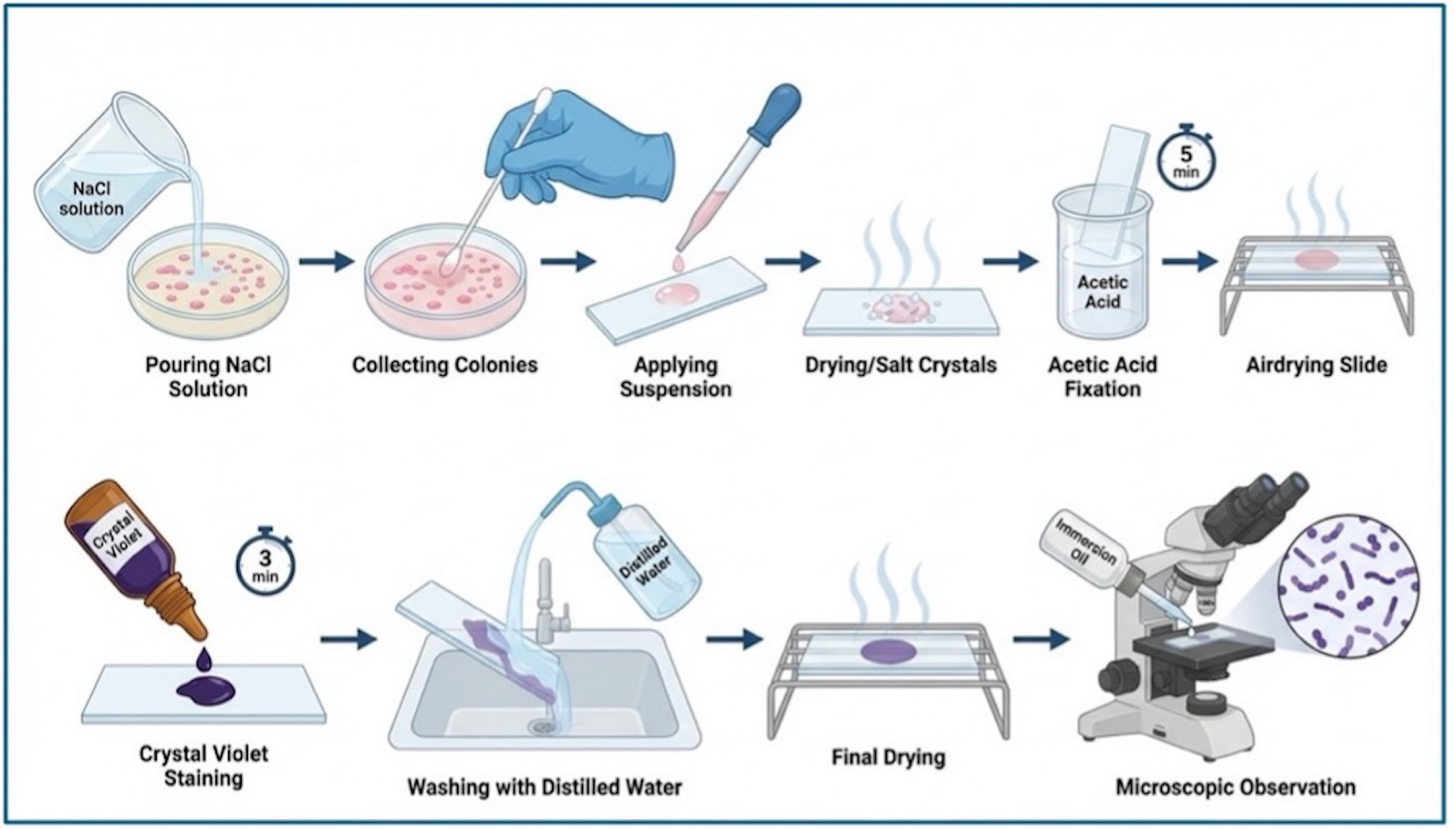
Preparation of microscope slides for *H. salinarum* observation

Procedure (see Fig. 2.)

1. Pour a few millilitres of the NaCl solution into a Petri dish containing well-developed colonies.
2. With a cotton bud, remove the colonies by gently rubbing to obtain a cell suspension. This suspension should not be too thick. A slightly pink-coloured liquid is sufficient.
3. Drop a small drop of this microbial suspension on a microscope slide.
4. Let this drop dry out; salt crystals will precipitate.
5. Dip the slide in the acetic acid solution for 5 min; this step fixes the cells and dissolves the salt.
6. Remove the slide from the acid bath and let it air dry.
7. Put a drop of crystal violet on the preparation and let it rest for 3 min.
8. Remove the stain by thoroughly washing with distilled water. Refer to Section 13 (Disposal Considerations) of crystal violet’s Safety Data Sheet (SDS) before discarding the washing waste.
9. Let the slide dry.
10. Observe under the microscope without a cover slip at a 1,000× magnification with immersion oil.

Warning! It is recommended to collect the crystal violet washing liquid in a suitable container rather than pouring it directly down the sink.

## Results and observations

### Activity 1

The appearance of colonies becomes detectable after 2–3 days of incubation. Well defined pink coloured microbial structures appear after about 1 week.

**Fig. 3.**
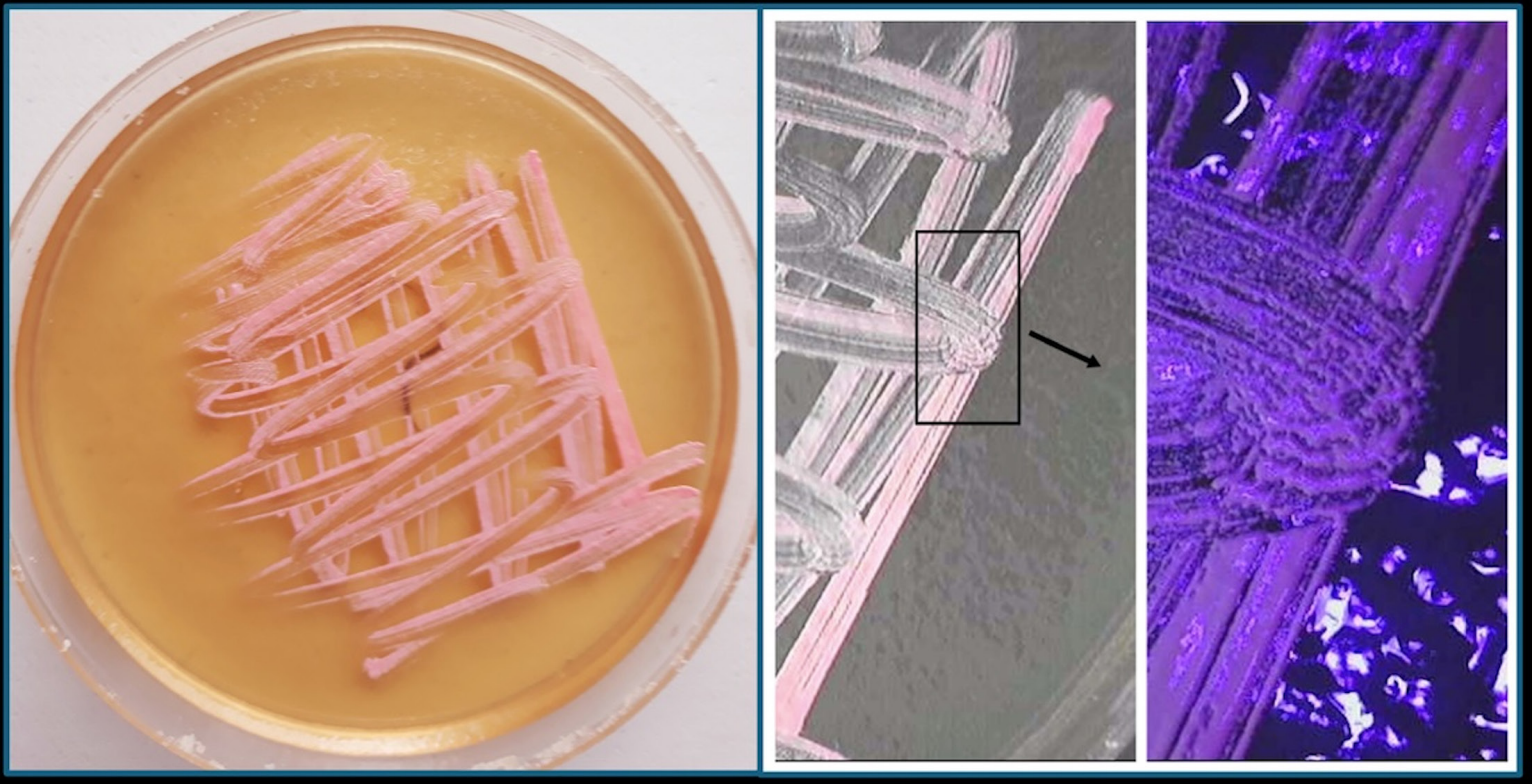
Appearance of *H. salinarum* colonies under visible and UV light

Under UV light, the colonies brightly fluoresce in purple (Fig. 3). This phenomenon is due to the presence of bacteriorhodopsin in the cell membranes of *H. salinarum*.

**Fig. 4.**
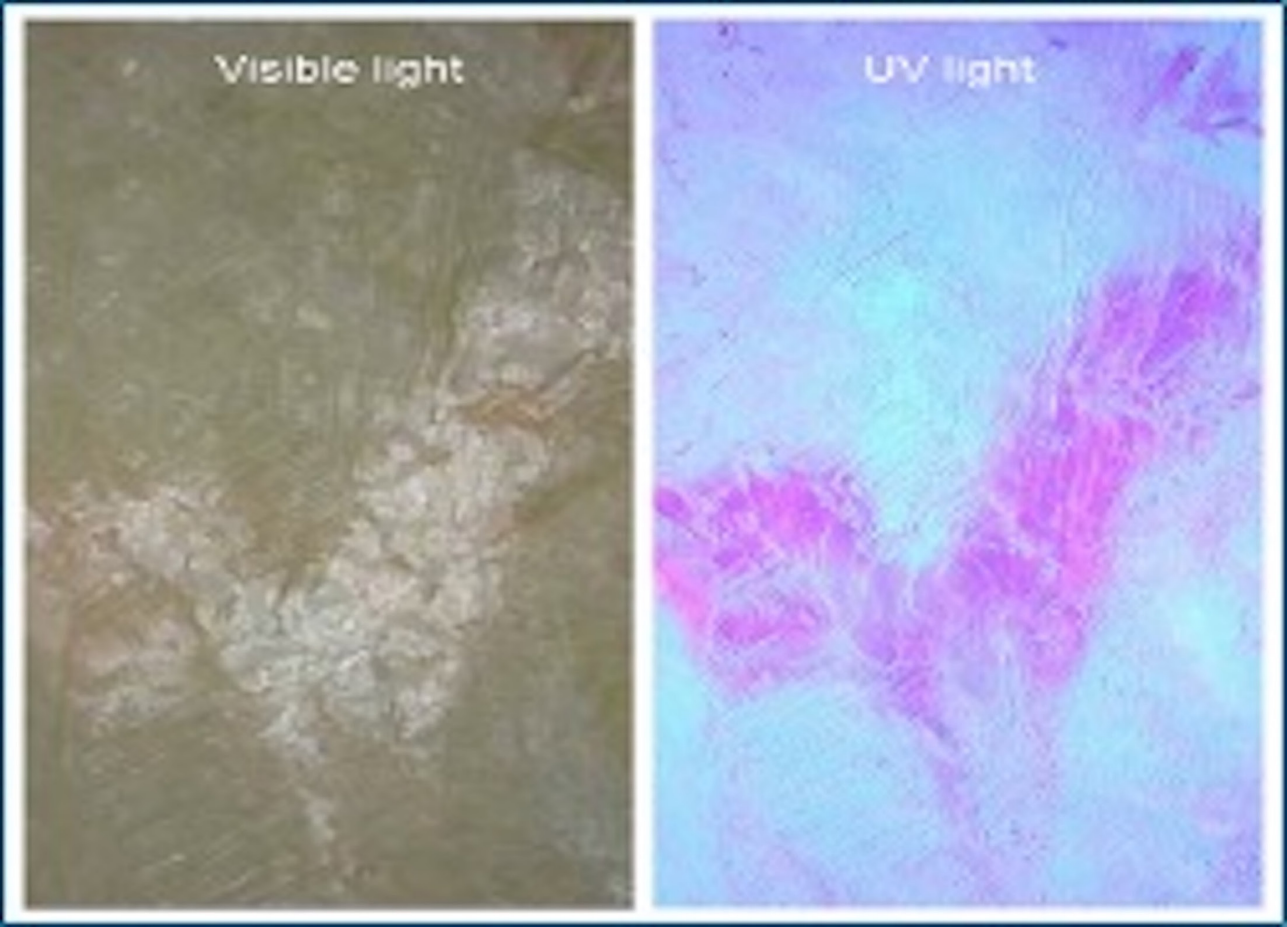
Embedded colonies in salt crystals

When the incubation time increases, salt crystals start precipitating inside the agar where microbial colonies may become embedded. If Petri dishes are left unattended for a long time, the whole culture dries out but, surprisingly, *H. salinarum* cells stay alive and can be subcultured. Fig. 4 shows a cluster of cells embedded in crystallized salt both under visible and UV light.

### Activity 2

**Fig. 5.**
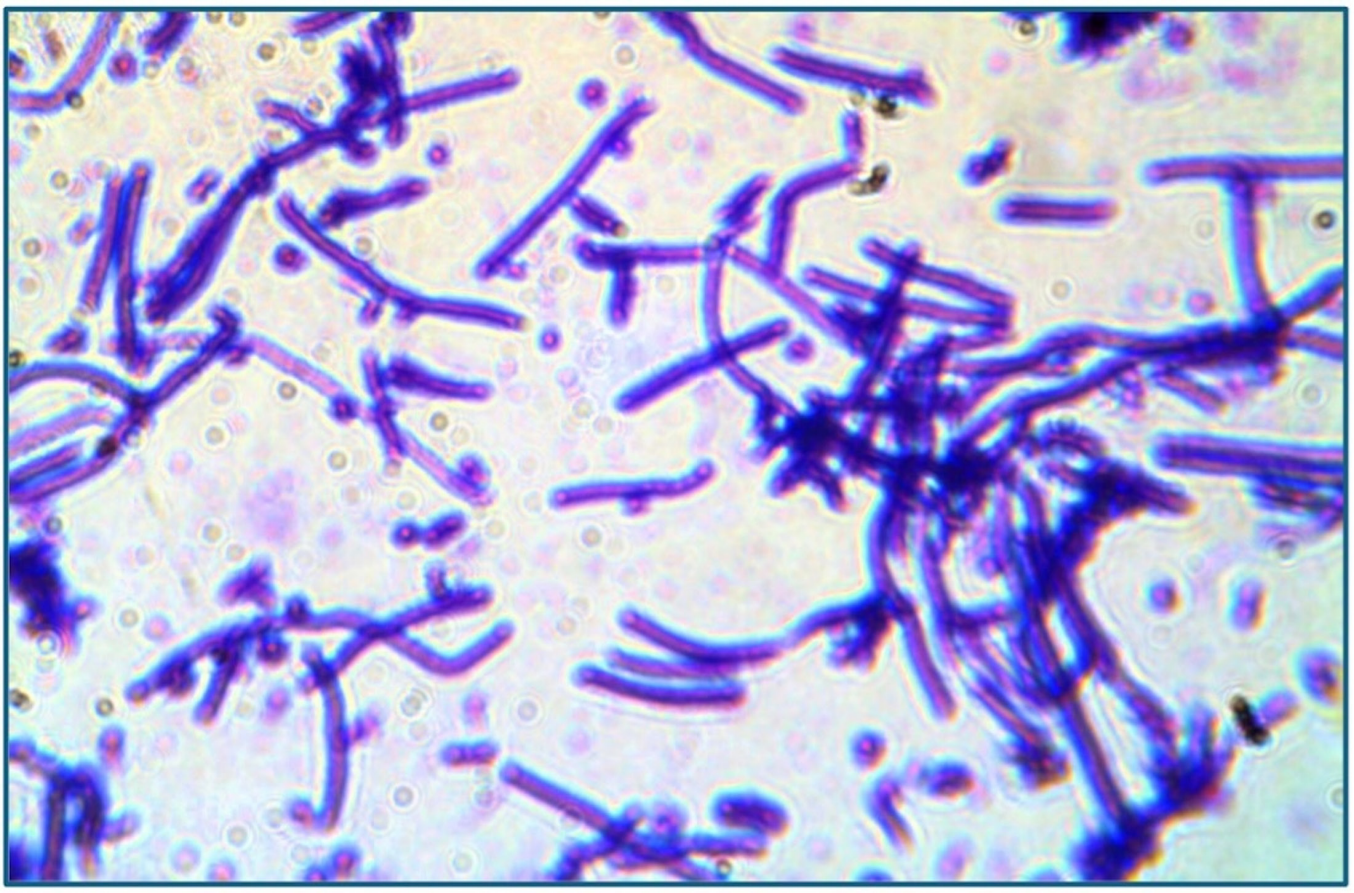
Crystal violet staining of *H. salinarium* cells (1,000×).

Cells are clearly visible and show a deep violet colour (Fig. 5). Cells are rod-shaped and appear flexible due to the absence of a cell wall (magnification: 1,000×).

## Discussion

In all respects, *H. salinarum* is unique and raises great interest.

In order to provide informed responses to the various questions outlined below, both instructors and students are strongly encouraged to consult the seminal article by Das Sarma *et al*. [4]. In addition, the website of this author’s laboratory offers an extensive collection of data and resources related to *Haloarchaea* [8]. With regard to applications, some companies have specialized in the production of by-products derived from the large-scale cultivation of *H. salinarum* [9]. More specifically, the bacteriorhodopsin of *H. salinarum* is employed in bioelectronics, a highly advanced and cutting-edge field of research [10].

The following questions may initiate further research with students.

Activity 1:

- Maybe this was your first contact with microbiology. What did you learn?
- Microbes have lots of applications (food technology, medicine, ecology etc.). Does *H. salinarum* have specific applications?
- Why are halophilic archaea considered one of the first lifeforms on Earth?
- What is fluorescence? Why do *H. salinarum* cells fluoresce?

Activity 2:

- Why do you have to suspend *H. salinarum* cells in a 20% salt solution?
- Why is it necessary to stain the cells?
- Have you ever heard about the Gram stain? What is the difference with the present protocol?
- What is the procedure to reach a magnification of 1,000×?
- Who invented the first microscope?

## Conclusion

These few simple experiments, which are very similar to those carried out in a ‘real’ microbiology laboratory, show that it is possible, apart from working under sterile conditions, to acquire basic skills in handling micro-organisms. Moreover, using *H. salinarium*, an archaeon, arouses curiosity because it is an *extremophilic* micro-organism capable of living in very specific biotopes: halophilic bacteria have even been discovered in salt crystals dating back to the Permian period, 250 million years ago [11, 12]! Another point of interest: halophilic microorganisms could potentially survive on other planets [13]. The present work has not been implemented under classroom conditions. As part of the future development of pedagogical activities, such implementation is planned, together with the collection of feedback from participants in order to optimize the teaching of microbiology.

## Abbreviations

SDS: safety data sheet

## References

[1] European Union (2000). Directive 2000/54/EC.

[2] UK Statutory Instruments 2002 No. 2677 (Schedule 3) (2002). The Control of Substances Hazardous to Health Regulations.

[3] WHO Team Biosecurity & Health Security Protection (BSP) (2020). Laboratory Biosafety Manual.

[4] Dassarma P, Tuel K, Nierenberg SD, Phillips T, Pecher WT (2016). Inquiry-driven teaching & learning using the archaeal microorganism *Halobacterium* NRC-1. Am Biol Teach.

[5] Eichler J (2023). *Halobacterium salinarum*: Life with more than a grain of salt. Microbiology.

[6] Lorentzen G, Wesmajervi Breiland MS, Østli J, Wang-Andersen J, Olsen RL (2015). Growth of halophilic microorganisms and histamine content in dried salt-cured cod (*Gadus morhua* L.) stored at elevated temperature. LWT - Food Sci Technol.

[7] Wikipedia Bacteriorhodopsin. https://en.wikipedia.org/wiki/Bacteriorhodopsin.

[8] Halo-Ed Halo-Ed Portal: The DasSarma Group Virtual Lab. https://halo-ed.org.

[9] HALOTEK HALOTEK Biotechnologie GmbH. https://halotek.de.

[10] Yu-Tao L (2018). A Review on Bacteriorhodopsin-Based Bioelectronic Devices. Sensors.

[11] Jaakkola ST, Ravantti JJ, Oksanen HM, Bamford DH (2016). Buried alive: microbes from ancient halite. Trends Microbiol.

[12] Vreeland RH, Rosenzweig WD, Powers DW (2000). Isolation of a 250 million-year-old halotolerant bacterium from a primary salt crystal. Nature.

[13] DasSarma S (2006). Extreme Halophiles Are Models for Astrobiology. Microbe Magazine.

